# Mitogenomic architecture of the multivalent endemic black clam (*Villorita cyprinoides*) and its phylogenetic implications

**DOI:** 10.1038/s41598-020-72194-1

**Published:** 2020-09-22

**Authors:** Summaya Rahuman, N. S. Jeena, P. K. Asokan, R. Vidya, P. Vijayagopal

**Affiliations:** 1 Marine Biotechnology Division, Central Marine Fisheries Research Institute, Kochi, 682 018 Kerala India; 2 Molluscan Fisheries Division, Central Marine Fisheries Research Institute, Kochi, 682 018 Kerala India; 3 Mangalore University, Mangalagangotri, Mangalore, 574 199 Karnataka India

**Keywords:** Evolution, Genetics

## Abstract

The Indian black clam *Villorita cyprinoides* (Family: Cyrenidae), an extractive commercially exploited species with aquaculture importance contributing more than 70% of clam fishery in India, is endemic to the Indian peninsula. Currently, there is very sparse information, especially on the molecular data of *Villorita*. The present study aims to provide a comprehensive knowledge of mitogenome architecture and assess the phylogenetic status of Cyrenidae. This has resulted in reporting the first complete mitogenome of *V. cyprinoides* using next-generation sequencing technology. The A+T circular mitogenome was 15,880 bp long, exhibiting 13 protein-coding genes (PCGs) including ATP8 (absent in several bivalves), 22 transfer RNA, and two ribosomal RNA genes residing in the heavy strand in a clockwise orientation and a gene order akin to *Corbicula fluminea*. The molecular phylogeny inferred from a concatenated multi-gene sequence [14 mitochondrial (12 PCGs, *rrn*S and *rrn*L) and two nuclear genes (Histone H3, 18S rRNA)] from 47 representative species of superorder Imparidentia, clustered *V. cyprinoides* and Cyrenid clams to a single clade supporting the monophyly of Cyrenidae. The subsequent mitochondrial gene order analysis substantiates the close relationship of *V. cyprinoides* and *C. fluminea,* analogous to phylogenetic output. The multilocus tree topology calibrated with verified fossil data deciphered the origin and diversification of Cyrenid clams during late Triassic-early Jurassic. The data derived from this study shall contribute remarkably for further insights on cryptic species identification, molecular characterization of bivalve mitogenomes and mitochondrial evolutionary history of genus *Villorita*. Moreover, complete mitogenome can aid in potential marker development for assessing the genetic health of black clam populations.

## Introduction

In 2018, global molluscan aquaculture production was 17.7 million tons^1^, elucidating its astounding potential as a food resource of international importance. The practice of inclusion of bivalves in the diet has had an exquisite role in human ethos for ages^2, 3^. The dependence on bivalves for food, industries including jewellery, medical, and ecosystem services challenged the scientific community to enrich the information on various aspects including phylogeny, evolutionary history, genetic improvement and breeding of superior strains for enhancing aquaculture production^4–6^.

Mitochondrial DNA (mt-DNA), the lone double-stranded circular extranuclear genome in eukaryotes prevailed by endosymbiosis, is of crucial importance because of its ATP synthesis in the cell through oxidative phosphorylation^7, 8^. Besides energy production, small size, high copy number, conserved genes, high rate of evolution, low recombination and maternal inheritance enables mitochondria as a potential marker in molecular studies^8, 9^. Bivalve mitogenome as metazoans typically consists of 13 protein-coding genes for OXPHOS, 2 rRNA, 22 tRNAs and an A+T rich region controlling replication and transcription^10^. However, peculiar mitochondrial features like extensive genome reorganisation and occurrence of Doubly Uniparental Inheritance (DUI) in certain bivalves extended supplementary information on evolutionary patterns across species and among conspecifics^11–13^. The popularity of mitogenome as a marker of choice for phylogenetic studies is on arise due to a surge in published mitogenomic data which provides improved phylogenetic resolution^14^. Nevertheless, molecular studies employing combined mitochondrial and nuclear markers played a pivotal role in imparting accurate and refined knowledge on bivalve molecular systematics, phylogenetics, population health analysis and comparative phylogenomics^15, 16^_._

**Figure 1:**
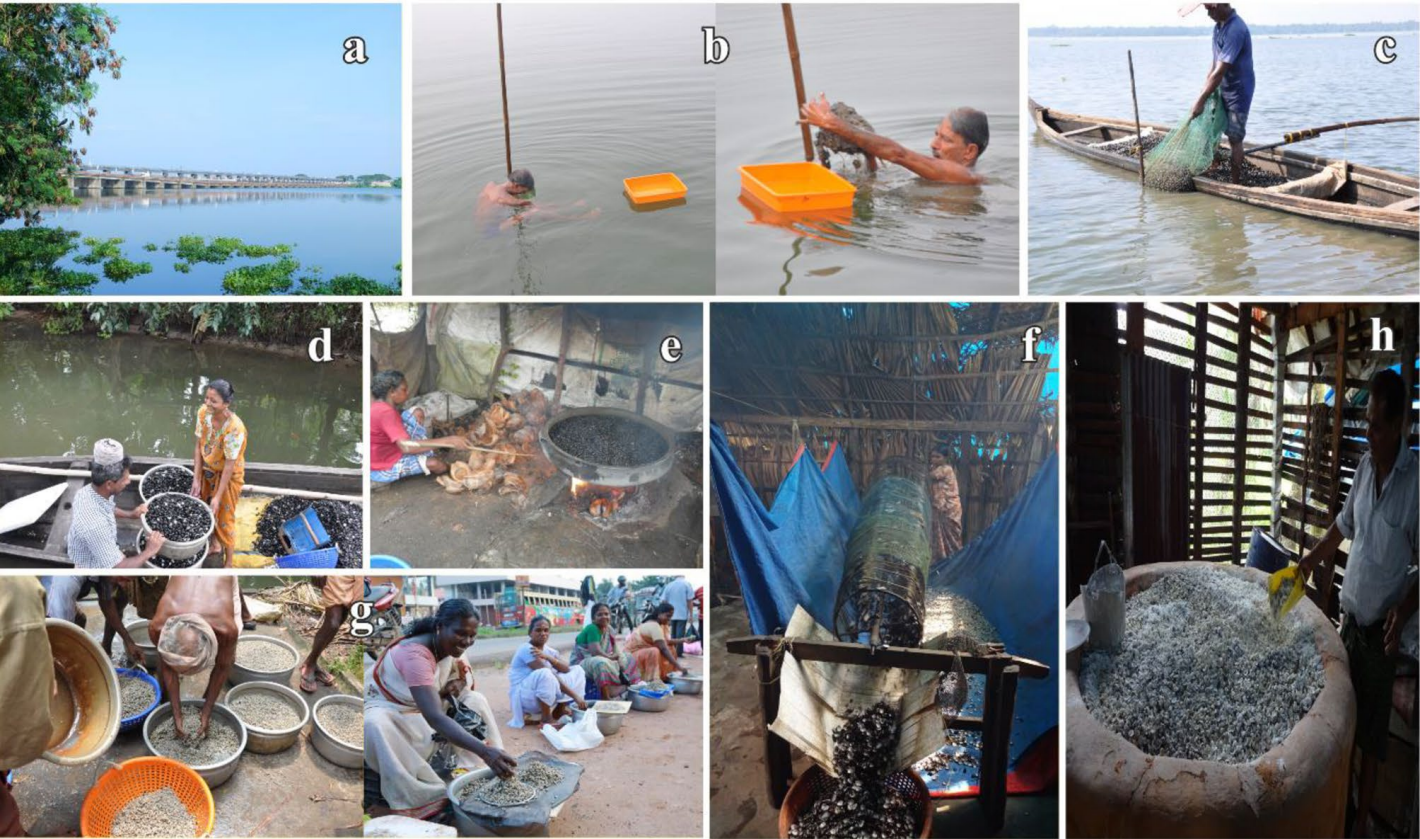
**(a**) The Thanneermukkom Barrage dividing the Vembanad Lake to Northern and Southern regions. (**b**) Clam collection by diving and handpicking. (**c**) Collection using *kolli*, an indigenous hand rake net. (**d**) Transportation of collected clams to shore. (**e**) Meat shucking by cooking the clam. (**f**) Separation of clam meat by sieving. (**g**) Marketing of clam meat. (**h**) Shells of black clam burn in a specially built kiln for manufacture of cement. Photo courtesy to Jeena N. S (**a**, **g**), Vidya R (1**b**–**d**) and Asokan P. K (**e**, **f**, **h**) of CMFRI, Kochi, Kerala. The images were processed manually in Adobe Photoshop CC, 2020.

*Villorita cyprinoides* (Gray,1825) (Indian black clam), a bivalve belonging to family Cyrenidae inhabiting on or even below the surface of bottom sediment in the brackish water ecosystem, is endemic to the peninsular India^17^. Cyrenidae constitutes a diverse range of species with differing attributes (e.g. economically important along with invasive taxa) is known for retention of brackish water genera and parallel radiation of species to freshwater and brackish water environments^18^. Even though the brackish water biome is the natural habitat of *V. cyprinoides*, wide salinity tolerance of this resilient species enables its range expansion to the freshwater environment^19^, as exemplified in Vembanad Lake (Kerala, India) seasonally divided by the Thanneermukkom Barrage (Fig. 1a). The genus *Villorita* embracing *V. cyprinoides* is traditionally considered to be monotypic^20^ and acclaimed as prime species in Indian clam fisheries, prominently cherished for both meat and shell^21^. Despite the tedious collection and processing exercise for the marketing of black clam (Fig. 1b–g), the species always maintains a supreme demand in the market due to its nutritive value^22^. The use of the species for local consumption, export and protein supplement in shrimp and poultry feed, makes the natives depend mainly on the clam for their sustenance and livelihood^23^. The calcium-rich shell, a by-product in clam fishery is a potential raw material for the manufacture of lime and cement^21^ (Fig. 1h). The recent study which revealed the presence of antioxidative and anti-inflammatory metabolites from *V. cyprinoides*^24^ opens up new avenues of research in nutraceuticals. Apart from the top-notch economic significance, bioremediation, biocontrol of phytoplankton as well as biomonitoring of estuarine heavy metal pollution is feasible through *Villorita*^25, 26^.

*Villorita cyprinoides* distributed in Vembanad Lake, a Ramsar site and longest lake in India, contribute 88.2% to the black clam fishery of the country^27^. The lake is crossed by the Thanneermukkom Barrage constructed (in 1974, ~ 2 km in length) to prevent the intrusion of saltwater, a potential threat to the rice production in Kuttanad paddy fields (~ 50,000 acres lying south to Vembanad Lake) known for the unique practice of rice cultivation below sea level (Fig. 1a). The barrage divides the lake to a northern side with flourishing beds of black clam and a southern part with a diminishing clam population. The reproductive success of the black clam depends on salinity and the decline in the clam population on the southern side may be correlated with the drop in salinity associated with closure (in December–May) and opening of the barrage during monsoon creating a freshwater environment. Further, flooding results in the burial of clam under silt and clay carried by floodwater and washing off the natural beds creating ecological changes^28^. The imbalanced ecosystem created through the construction of barrages results in restricted gene flow within conspecifics^29^. The detrimental effect of the decline in black clam population in the southern side of the Vembanad Lake affecting the livelihood of people depending on clam fishery urged the Govt. of Kerala to take immediate actions for rehabilitation through clam relaying (G.O(Rt)No. 460/16/F&PD of Fisheries and Ports (C) Department, Thiruvananthapuram, dated 01.08.16) to replenish the stock so as to ensure a sustainable supply of food, as implemented in many developed countries^30, 31^. Also, the dredging for the sub-fossil deposit of clamshell for industrial use^32^, pollution due to effluents from shrimp processing plants, eco-tourism, urban and agricultural runoff and infestation of the invasive *Eichhornia crassipes* engender threats to the clam^33^ in its natural habitat.

The economic and ecological eminence of *Villorita cyprinoides* results in continuous demand leading to overexploitation of the species including juveniles. Extensive harvesting of clam from the wild, niche alterations and exploitation of juveniles could be a potential threat to the existence of the species^34^. The selective pressure applied in stock by overfishing cannot be reversed through genetic selection on phenotypic traits^35^. In-depth studies on the exploitation of juvenile black clams suggested implementation of Minimum Legal Size (MLS) in clam fishery to reduce economic loss and preserve genetic variance^36^. The ecosystem degradation as well as overexploitation, if continued at the present level, may result in the reduction of effective population size leading to increased homozygosity, reduction in fitness due to faster erosion in genetic variation of related traits and ultimate genetic drift in far or near future. Along with genetic information, the need for breeding standardisation and proper farming technique is inevitable for a sustainable black clam fishery.

*Villorita cyprinoides,* a resource of paramount importance in meeting the country’s need for a cheap and balanced diet, has received less attention at the genetic level. The present status of black clam fishery demands genetic investigation and analysis for the proper implication of management and conservation. The present work is a genuine attempt to determine the mitogenome architecture of *V. cyprinoides* thereby delivering ample molecular data for genus *Villorita.* The multi-gene phylogeny from mitochondrial and nuclear genes was attempted to allocate *V. cyprinoides* in the phylogenetic network and resolving the phylogenetic status of Cyrenidae. Additionally, mitochondrial gene order (GO) analysis and estimation of divergence time were also inferred using multiple genes and fossil data.

## Results and discussion

### Mitogenome characteristics

**Figure 2:**
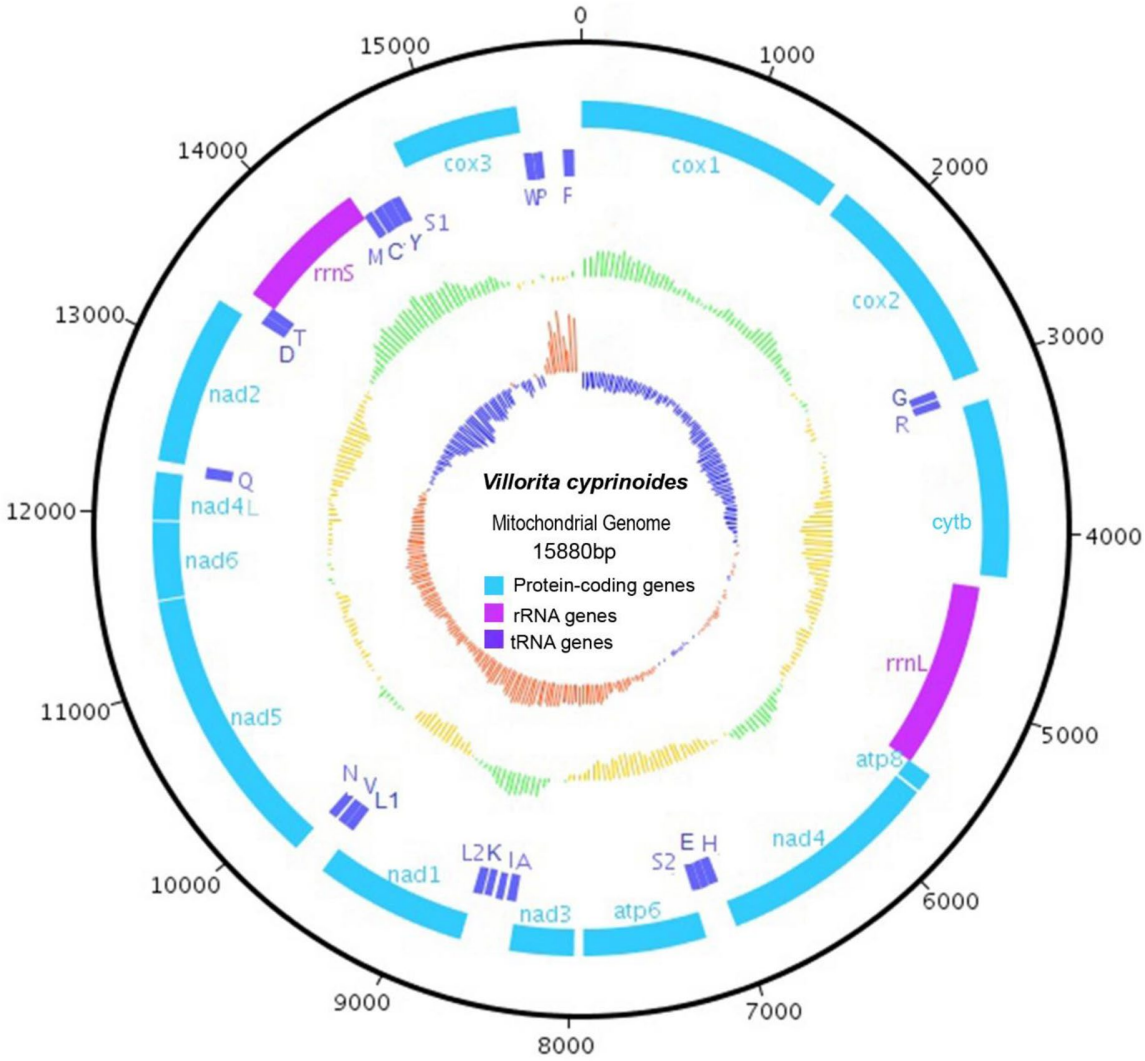
Mitochondrial genome organisation of *Villorita cyprinoides* visualised with DNA Plotter. The outer black circle represents the complete mitogenome of *V. cyprinoides* (scale denoted in base pair). The second ring represents mitochondrial proteins encoding genes, and the third circle denotes both rRNAs and tRNAs wherein all genes predicted in a positive frame. Colour in the box codes for specific categories of mitochondrial genes that are abbreviated according to standard usage. Fourth circle represents the distribution of GC % following yellow colour as below average and green colour, above-average values. The inner-circle represents the distribution of GC skew %, displaying blue colour as below average and red colour, above-average values.

The female mt-genome of *Villorita cyprinoides* is 15,880 bp in length, comparable to previously reported bivalve mitogenomes. Gene content of *V. cyprinoides* displayed 37 typical sets of genes found in metazoan mitogenome^10^ constituting 13 protein-coding genes (PCGs); encoding three electron transport chain (ETC) complexes (nd1-nd6, nd4l, cytb and cox1-cox3) and F_0_F_1_ ATP synthase complex (ATP6 and ATP8) in OXPHOS. Besides PCGs two ribosomal RNA (rRNA) and 22 transfer RNA (tRNA) genes were observed with the putative control region (CR). All the 37 genes are located on major (H-strand) strand with forward orientation like in other Heterodont bivalves^37^. Figure 2 plots the circular map of black clam mitogenome.

The base composition of the mt-genome holds A = 25.1%, T = 42.9%, G = 22.8%, C = 9.2% with A+T content representing 68% of the total genome. A positive GC skew (0.424) and negative AT skew (− 0.262) were noticed indicating a bias towards Ts and Gs. Comprehensive analysis of mitogenomes, PCGs and rRNAs amongst 47 species from orders Veneroida, Myida, and Cardiida disclosed rich A+T content and low C content along with positive GC and negative AT skew (Supplementary Table S1).

### Protein coding genes

All the 13 PCGs reported in *V. cyprinoides* mitogenome were identified within 11,835 bp length (74.5% of mitogenome), and A+T content predicted as 67.5% ranging from 63.8% (nad1) to 74.4% (nad4l). PCGs together encode 3,932 codons including nad5 (558aa) as the longest protein-coding gene and ATP8 (37aa) as the shortest. Nucleotide bias between all PCGs was estimated through base skewness (Table 1).

**Table 1:** Base composition, AT skew, and GC skew of the whole mt-genome, PCGs, rRNA, and tRNA genes of *Villorita cyprinoides*.

Region	A (%)	T (%)	G (%)	C (%)	GC (%)	AT (%)	GC Skew G−C/G+C	AT skew A-T/A+T
Complete mitogenome	25.1	42.9	22.8	9.2	32.0	68	0.42	− 0.26
PCGs	22.5	45	23.4	9.1	32.5	67.5	0.32	− 0.33
rRNA	33.6	35.9	20.8	9.7	30.5	69.5	0.36	− 0.03
tRNA	31.6	37.4	20.7	10.3	31	69	0.34	− 0.08
cox1	22.6	42.3	23.7	11.5	35.2	67.8	0.35	− 0.3
cox2	26.1	39.7	26.3	7.8	34.2	65.8	0.54	− 0.2
cox3	19.4	45.1	24.9	10.7	35.5	64.5	0.4	− 0.4
cytb	22.1	46.0	20.1	11.8	31.9	68.1	0.26	− 0.35
nad1	19.8	44.0	26.8	9.4	36.2	63.8	0.48	− 0.38
nad2	20.4	49.7	24.0	5.9	29.9	70.1	0.6	− 0.42
nad3	26.2	46.8	19.8	7.1	27.0	73	0.47	− 0.28
nad4	21.9	45.5	23.6	8.9	32.5	67.5	0.45	− 0.35
nad4l	18.6	55.8	21.1	4.6	25.6	74.4	0.64	− 0.5
nad5	24.4	44.8	21.4	9.4	30.8	69.2	0.39	− 0.29
nad6	21.7	43.3	26.8	8.3	35.0	65	0.53	− 0.33
ATP6	23.2	48.3	20.9	7.6	28.5	71.5	0.47	− 0.35
ATP8	21.1	49.1	21.9	7.9	29.8	70.2	0.47	− 0.4
*rrn*L	34.2	37.3	19.4	9.1	28.6	71.4	0.36	− 0.04
*rrn*S	32.9	33.9	22.6	10.6	33.2	66.8	0.36	− 0.01

Most of the PCGs employ ATN (ATG, ATA and ATT) as an initiation codon. In addition to the classical metazoan start codon, GTG (Valine) initiated translation^38–40^ of ATP6 and nad2 gene in Cyrenid clams^41^ including *V. cyprinoides*. Ten PCGs were terminated by TAG, and TAA codons whilst remaining were terminated by truncated codons T– and TA- which presumably functions as complete termination codon after post-transcriptional polyadenylation^42, 43^ (Table 2).

**Table 2:** General mitogenome characteristics of *Villorita cyprinoides*.

Gene	Start position	Stop position	Gene length	Start codon	Stop codon	Anticodon
Cytochrome c oxidase subunit 1	1	1,614	1,614	ATT	TAA	
Cytochrome c oxidase subunit 2	1,696	3,012	1,317	ATG	TAG	
tRNA-Gly	3,027	3,089	63			TCC
tRNA-Arg	3,093	3,154	62			TCG
Cytochrome b	3,188	4,315	1,128	ATA	TAG	
16 s ribosomal RNA	4,339	5,521	1,183			
ATP synthase F0 subunit 8	5,525	5,638	114	ATG	TAG	
NADH dehydrogenase subunit 4	5,645	7,002	1,358	ATT	TA-	
tRNA-His	7,003	7,064	62			GTG
tRNA-Glu	7,064	7,127	64			TTC
tRNA-Ser2	7,123	7,185	63			TGA
ATP synthase F0 subunit 6	7,186	7,926	741	GTG	TAA	
NADH dehydrogenase subunit 3	7,976	8,368	393	ATA	TAG	
tRNA-Ala	8,375	8,438	64			TGC
tRNA-Ile	8,456	8,519	64			GAT
tRNA-Lys	8,534	8,596	63			TTT
tRNA-Leu2	8,598	8,663	66			TAA
NADH dehydrogenase subunit 1	8,663	9,573	911	ATG	TA-	
tRNA-Leu1	9,574	9,637	64			TAG
tRNA-Val	9,637	9,701	65			TAC
tRNA-Asn	9,707	9,770	64			GTT
NADH dehydrogenase subunit 5	9,771	11,477	1707	ATG	TAA	
NADH dehydrogenase subunit 6	11,480	11,950	471	ATG	TAG	
NADH dehydrogenase subunit 4L	11,960	12,244	285	ATA	TAA	
tRNA-Gln	12,246	12,313	68			TTG
NADH dehydrogenase subunit 2	12,314	13,339	1,026	GTG	TAG	
tRNA-Asp	13,348	13,413	66			GTC
tRNA-Thr	13,418	13,480	63			TGT
12 s ribosomal RNA	13,483	14,343	861			
tRNA-Met	14,344	14,412	69			CAT
tRNA-Cys	14,415	14,481	67			GCA
tRNA-Tyr	14,487	14,552	66			GTA
tRNA-Ser1	14,552	14,620	69			TCT
Cytochrome c oxidase subunit 3	14,729	15,494	766	ATG	T–	
tRNA-Trp	15,495	15,556	62			TCA
tRNA-Pro	15,557	15,619	63			TGG
tRNA-Phe	15,767	15,831	65			GAA

ATP8 gene, which lacks in most of the heterodont bivalves was identified in *V. cyprinoides*, encoding a protein of 37 amino acids. No putative conserved domains were detected by pBLAST, whereas conserved amino acid block (WW**KR*Y*F) were identified as in certain species in family Veneridae^44^ and Cardiidae^45^ (Supplementary Figure S2).

Following the Relative Synonymous Codon Usage (RSCU) value of *V. cyprinoides*, all the 62 codons (excluding stop codon, TAA and TAG) were utilised in unusual frequencies (Table 3). In *V. cyprinoides* third codon position (75.1) contains high A+T content compared to first (62.6) and second (64.6) position. Bias towards A+T in the third codon position might be interconnected with degeneracy^46^ or natural selection on Wobble nucleotide^47^. Codon usage bias indicates TTT (Phe) is the most likely used codon after GTT (Val) whereas, CTC (Leu), CAC (His), TGC (Cys) were least used. It is evident that frequently used codons are A+T rich; while the lowest used were abundant in G+C content. The commonly used amino acid is Leucine than Phenylalanine, while Glutamine is less used.

**Table 3:** Codon Usage Bias and RSCU of PCGs in *Villorita cyprinoides*.

Amino acid	Codon	Count	RSCU	Amino acid	Codon	Count	RSCU
Phenylalanine	UUU	423	1.92	Tyrosine	UAU	142	1.86
	UUC	17	0.08		UAC	11	0.14
Leucine	UUA	226	2.54	Stop codon	UAA	0	0
	UUG	204	2.3		UAG	0	0
	CUU	67	0.75	Histidine	CAU	64	1.78
	CUC	2	0.02		CAC	8	0.22
	CUA	24	0.27	Glutamine	CAA	18	0.75
	CUG	10	0.11		CAG	30	1.25
Isoleucine	AUU	243	1.95	Asparagine	AAU	84	1.77
	AUC	6	0.05		AAC	11	0.23
Methionine	AUA	122	1.07	Lysine	AAA	56	0.97
	AUG	106	0.93		AAG	59	1.03
Valine	GUU	247	2.4	Aspartic acid	GAU	83	1.93
	GUC	3	0.03		GAC	3	0.07
	GUA	90	0.88	Glutamic acid	GAA	41	0.67
	GUG	71	0.69		GAG	81	1.33
Serine	UCU	138	2.54	Cysteine	UGU	70	1.94
	UCC	8	0.15		UGC	2	0.06
	UCA	32	0.59	Tryptophan	UGA	59	1.04
	UCG	9	0.17		UGG	55	0.96
Proline	CCU	86	3.07	Arginine	CGU	20	1.27
	CCC	2	0.07		CGC	3	0.19
	CCA	15	0.54		CGA	19	1.21
	CCG	9	0.32		CGG	21	1.33
Threonine	ACU	77	2.77	Serine	AGU	73	1.35
	ACC	3	0.11		AGC	7	0.13
	ACA	21	0.76		AGA	91	1.68
	ACG	10	0.36		AGG	76	1.4
Alanine	GCU	104	2.62	Glycine	GGU	134	1.7
	GCC	5	0.13		GGC	19	0.24
	GCA	33	0.83		GGA	62	0.79
	GCG	17	0.43		GGG	100	1.27

### Ribosomal and transfer RNAs

**Figure 3:**
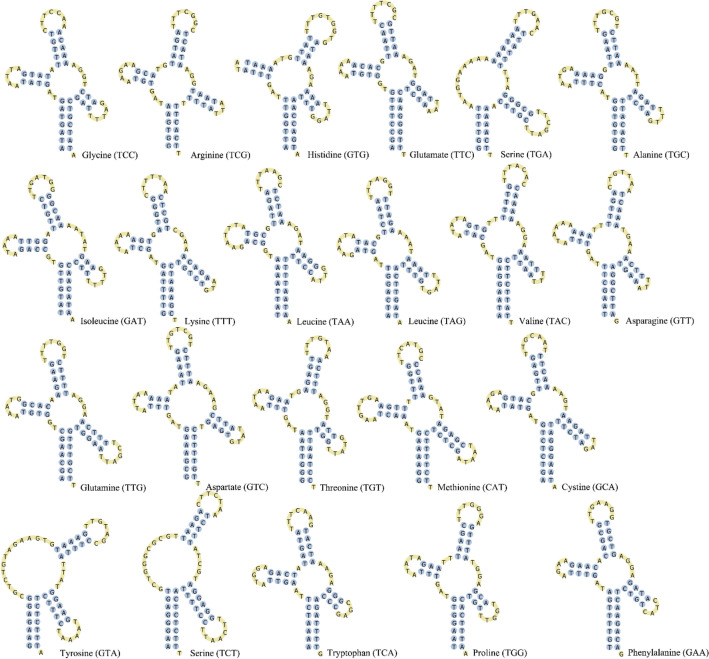
Cloverleaf secondary structure of 22 tRNA genes in *Villorita cyprinoides* mitogenome generated using PseudoViewer v.3.0 (https://pseudoviewer.inha.ac.kr/).

Twenty canonical tRNAs along with single gene duplication of *trn*S and *trn*L were bestrewed within the genome (8.9% of mitogenome). tRNAscan-SE identified only eleven tRNA gene whereas ARWEN detected all tRNA genes except *trn*H, predicted by position and sequence homology to *trn*H gene of *Corbicula fluminea* and *Arctica islandica*. Individual tRNA gene length varies from 62 bp (*trn*H, *trn*W) to 69 bp (*trn*S1, *trn*M) with anticodons identical to other heterodont bivalves. Separate anticodons encode for duplicated Serine (UCN, AGN) and Leucine (UUR, CUN) genes. All tRNAs can be folded to typical clover-leaf secondary structure except for *trn*S genes. The secondary structure of 22 predicted tRNA genes were summarised in Fig. 3.

The cloverleaf structure of tRNAs consists of 6–8 nucleotide pairs in aminoacyl acceptor stem, 5–6 pairs in anticodon and 4–6 pairs in DHU (dihydrouridine) and TψC stem with mismatched Watson–Crick base pairing. Anticodon of all tRNA genes was succeeded by Uracil and preceded by Guanine or Adenine in the anticodon loop of *V. cyprinoides*. The DHU stem of least conserved *trn*S1 and *trn*S2 genes^48^ were replaced by a large DHU loop, an accepted feature in molluscs^44, 49^. DHU stem of *trn*Y gene is reduced to one nucleotide pair.

BLAST searches confirmed MITOS annotated ribosomal RNA genes, *rrn*L and *rrn*S in *V. cyprionoides* with length 1183 bp and 861 bp respectively (12.8% of mitogenome). Large ribosomal RNA, considered as the largest rRNA locus (*rrn*L) in bivalves^50^ flanked between the genes cytb and ATP8 as in genus *Corbicula*, *Calyptogena*, *Cyclina*, *Dosinia* and *Meretrix* (except *Meretrix meretrix*). Also, the position of small ribosomal RNA (*rrn*S), between *trn*T and *trn*M gene was consistent with genus *Corbicula*, *Calyptogena*, *Cyclina*, and *Dosinia* (Fig. 2). The exact boundaries of rRNA genes can only be determined by transcript mapping. Therefore, the indefinite length of *rrn*S and *rrn*L were resolved by comparison of their sequence similarity with species in family Cyrenidae and Veneridae.

### Non-coding regions

A total of 590 bp intergenic spacer (IGS) sequences, sizes varying from 1 to 147 bp spreading across 23 regions were determined. All non-coding regions above 50 bp fold to stem and loop structure and the longest non-coding region flanked by *trn*P and *trn*F having 147 bp length fragment can be considered as control region (CR). The fragment between *trn*P and *trn*F form the most stable secondary structure with palindrome sequence, and AT content slightly above *Villorita* mitogenome, which are the general characters of control region^46, 51^ controlling replication and transcription of the genome (Supplementary Figure S3).

Five gene overlappings comprising 1–3 bp were noticed between *trn*H-*trn*E, *trn*L2-nad1, *trn*L1-*trn*V, *trn*Y-*trn*S1, and, *trn*E-*trn*S2.

### Gene rearrangement and synteny

**Figure 4:**
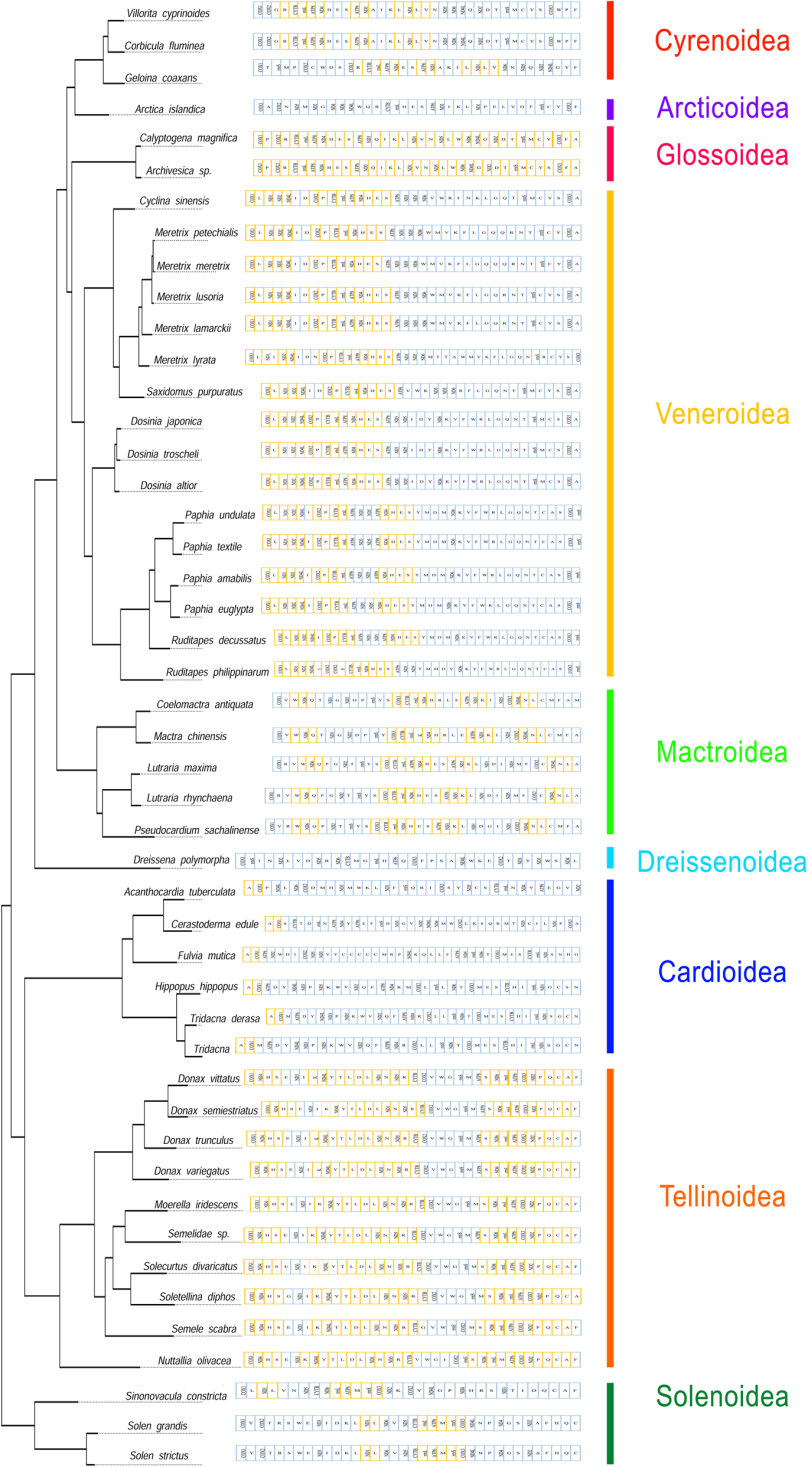
Linear illustration of Imparidentian mitogenomes superimposed on phylogenetic tree comparing synteny blocks in superfamily level. Tangerine colour bar depicts conserved gene order or synteny blocks**.**

Gene rearrangement, accepted as a marker of common ancestry^52^, varies unpredictably owing to single strand gene distribution in bivalves^50^. The gene rearrangement explained through TDRL (Tandem Duplication Random Loss) model is believed to transpire in bivalves as in vertebrates^53^. Though overall gene order is not well conserved within bivalve superfamilies, gene arrangement is substantially similar after excluding tRNA genes. Conversely, gene arrangements in Imparidentian bivalve families are nearly identical except for tRNA duplication, and translocation of genes, but variable between families. The conserved gene order or synteny blocks displayed in superfamilies are shown in Fig. 4.

The mitogenome order of bivalves in genus level alters dramatically^54^ albeit, comparative mt-genome analysis of Imparidentian clams revealed conserved gene order within the genus level. *V. cyprinoides* presented highly conserved synteny block with *Corbicula fluminea* for all 37 genes, but variable in gene length. Alternatively, species belonging to family Vesicomyidae (*Archivesica* sp. and *Calyptogena magnifica*) displays exact gene order as *V. cyprinoides* after removing tRNA genes though they represent a different family. These results reflect Vesicomid clams are related to family Cyrenidae than other families. Also, *V. cyprinoides* and *Corbicula fluminea* may be evolved recently and are closely related since gene rearrangement in closely related species is less than in distant^55^. The gene orders conserved in Cyrenid clams were identified as R-cytb-*rrn*L-ATP8-nad4, ATP6-nad3, and L-nad1-L-V. Species within genus *Donax*, *Solen*, *Dosinia*, shared identical gene order with less rearrangement like *Tridacna*, *Paphia*, and *Lutraria* (purging ATP8). The gene order within Cardioidea is highly rearranged and least conserved compared to other superfamilies.

### Phylogenetic analysis

**Figure 5:**
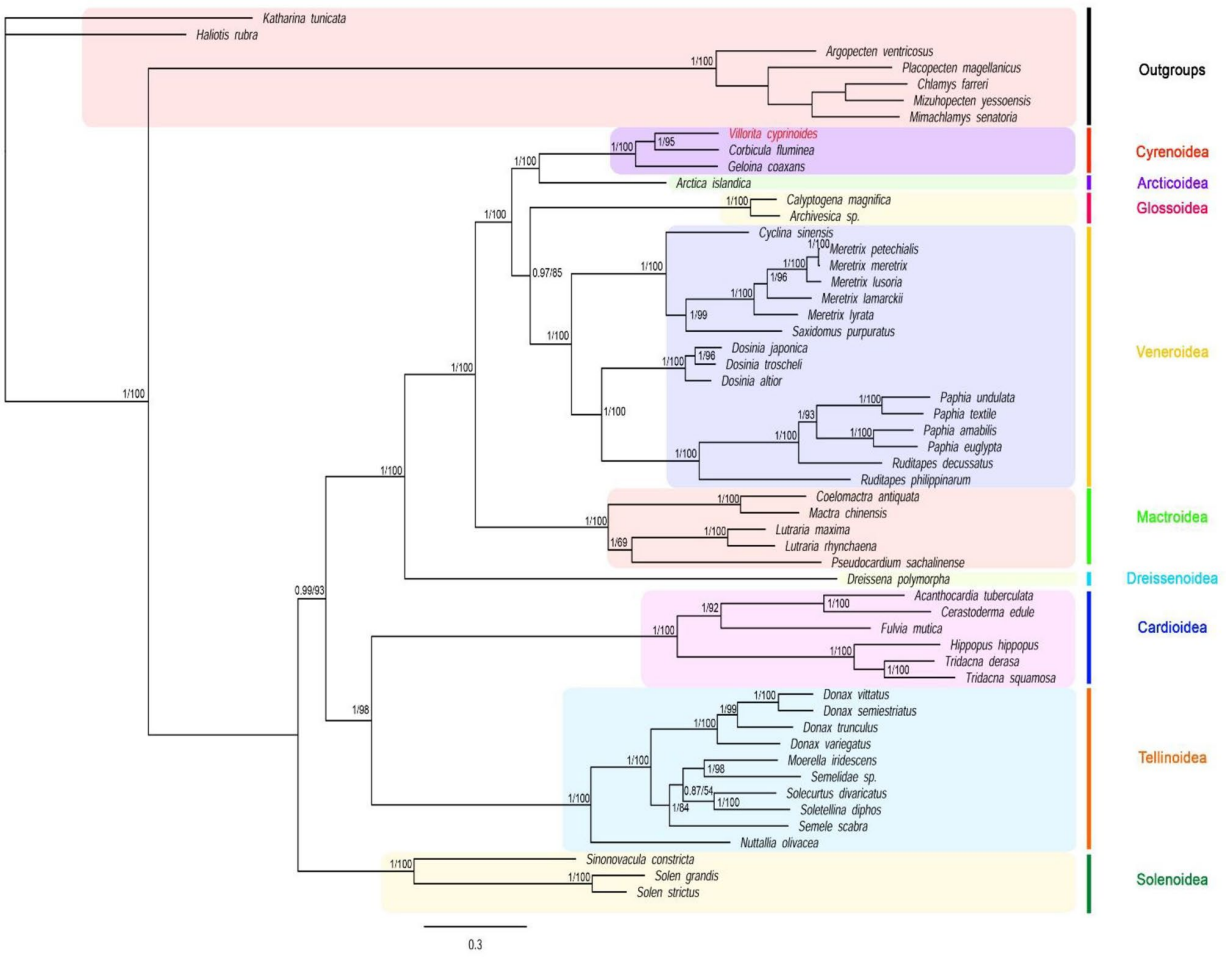
The Bayesian Phylogram evaluated based on nucleotide sequences of multilocus data (mitochondrial and nuclear) from 47 species of superorder Imparidentia. The ML tree exhibited identical topology. Node labels correspond to Bayesian posterior probability and ML bootstrap values and scale bar specify substitutions per site. Vertical coloured bar designate superfamilies.

Amplification of Histone H3 and small ribosomal rRNA (18S rRNA) nuclear genes resulted in a generation of 350 bp and 1725 bp amplicons respectively. The phylogenetic analysis was assessed after the exclusion of ambiguous loci using Gblock upon alignment. DNA sequences of mitochondrial (12 PCGs except for ATP8, *rrn*S and *rrn*L) and nuclear protein-coding (Histone H3), and non-coding genes (18S rDNA) after Gblock analysis were concatenated bestowing 11,767 base sequences for further phylogenetic reconstruction. The best partitioning scheme and best-fit substitution models were generated for concatenated multi-gene data providing 17 best subset partitions (Supplementary Table S4). Phylogenetic estimation incorporating the subset partitions and evolutionary models for both Maximum likelihood (ML) and Bayesian Inference (BI) analysis generated a well-resolved tree with similar topology. All major clades were well supported with posterior probability > 95 and bootstrap > 85. The combined tree with node label showing posterior probability (PP) values and bootstrap (BS) replicates was displayed in Fig. 5.

The phylogeny of Venerida, the most debated order in monophyletic subclass Heterodonta, is still undergoing extensive revision^56–59^. The present study considered 47 species, including 30 Venerida, 16 Cardiida, and one Myida confirmed the monophyly of the Venerid family, Cyrenidae^60^. Family Cyrenidae (Superfamily: Cyrenoidea) form a well-supported clade corroborating monophyly, placing *G. coaxans* towards the stem, and *V. cyprinoides* and *C. fluminea* to its crown. Cyrenidae co-clusters with Arcticoidae establishing Arcticoidae as immediate sister taxon, whereas Vesicomyidae (Glossoidea) lineage form a separate clade.

*D. polymorpha* (family: Dreissenidae), freshwater bivalve belonging to order Myida, found nested within Venerid taxa, questioning the phylogenetic status of the orders, and subdivision of Henterodonta to Venerida and Myida^61^. In contrast, this result substantiates the separate radiation of Cyrenidae and Dreissenidae to the freshwater lineage from marine ancestors^18^. The monophyletic status of Mactridae (Superfamily: Mactroidea) was confirmed, which appeared as a sister group to Dreissenidae^62^. Mactridae clustered with major clade [(Veneridae + Vesicomyidae) + (Cyrenidae + Arcticoidae)].

Phylogenetic analysis of Veneridae, the most diverse and economically important taxon in Venerida recovered the monophyletic status^63^ forming Vesicomyidae as a sister group. Although phylogenetic analysis suggests Vesicomyidae as a sister group, Veneridae shares close kin with Cyrenidae and Arcticoidae. The close relation of Veneridae with Vesicomyidae, Arcticoidae and Cyrenidae was supported by the presence of hinge characters, a morphological synapomorphy^64^. The major clade of Veneridae split separately, forming two distinct subclades retaining Cyclininae, Meretricinae, and Pitarinae in one clade, and Dosiniinae and Tapetinae subfamilies in another, agreeable with the previous studies^65^.

Furthermore, Pharidae and Solenidae supported as the sister group to each other, converged to form a well-resolved clade pointing to the monophyly of superfamily Solenoidea as expected^66^.

Being sister taxon to each other, Tellinoidea and Cardioidea validities the monophyly of order Cardiida. Five Tellinoidean families aggregated to a single clade with high PP and bootstrap value indicating monophyly of Tellinoidea but failed to resolve familial relationship^62^. However, species from Donacidae clustered to a branch, forming a sister group with other Tellinoid families^9^. Conversely, the monophyletic status of Carridae and Tridacnidae were evident in our phylogenetic analysis^67^, wherein Cardioidea itself is monophyletic.

The monophyletic status of Cardiida and Solenoidea were well supported, but we could not ratify the exact phylogenetic status of Tellinoidean families. Like Cardioidea and Solenoidea, the monophyly of Mactridae was validated, but recent studies support paraphyletic evolution^66^. This disparity might be due to limited taxon sampling because of less available mitogenome and nuclear gene data, exemplifying the importance of using multiple genes in phylogenetics.

### Divergence time estimation

**Figure 6:**
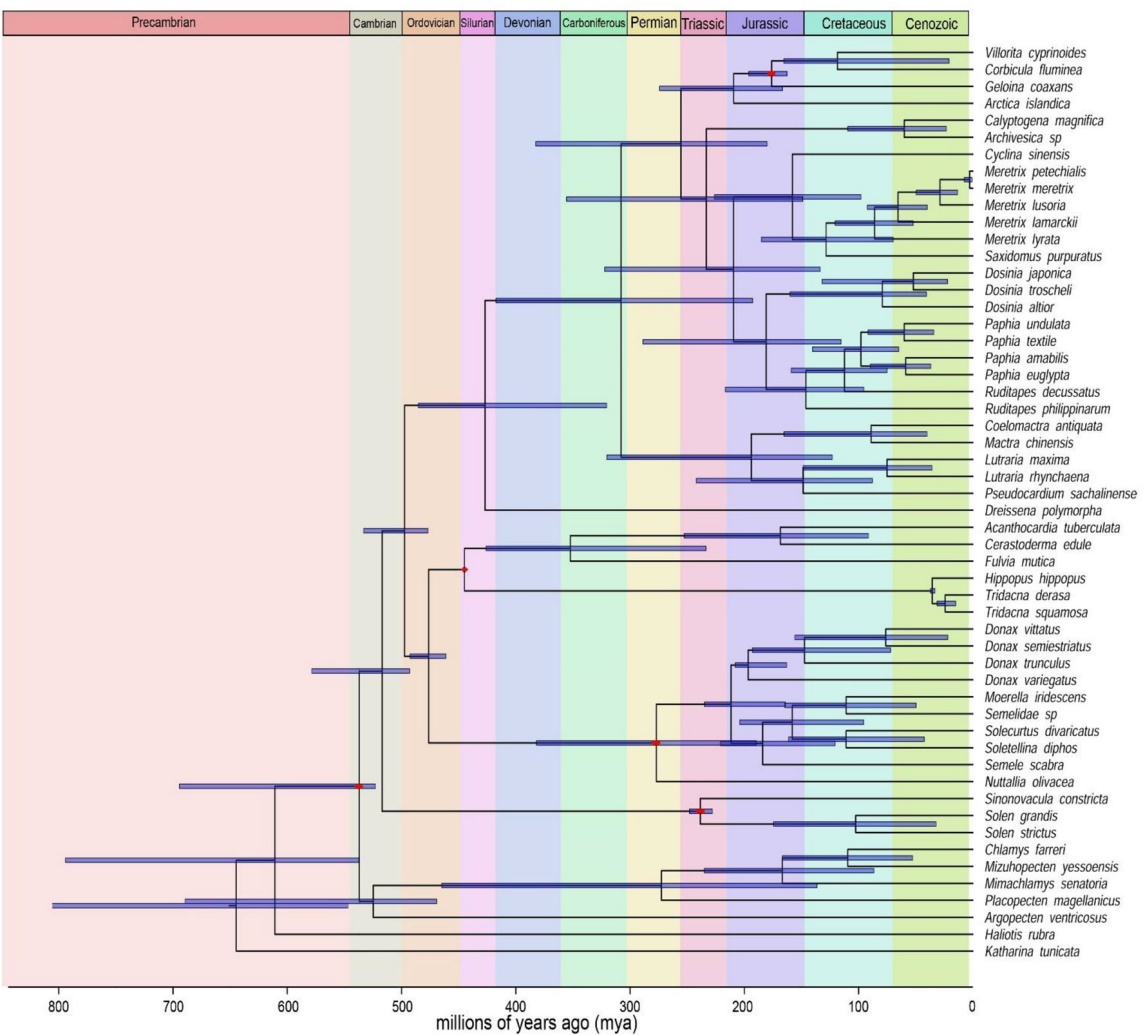
The time-calibrated multi-gene tree topology along with the geological time scale (mya). Red circles denote the fossil calibration points and horizontal bar indicate 95% HDP (highest posterior density) for node ages.

The multi-gene tree topology after phylogenetic analysis were employed for molecular dating. Evolutionary ages and divergence were estimated through calibration of the molecular clock using multiple fossil data (Table 4). The time-calibrated phylogenetic tree is shown in Fig. 6.

**Table 4:** Table showing incorporated fossil data for molecular divergence estimation.

Sl.no	Taxa	Clade upper	Clade lower	Geological time scale	Fossil datation (mya)	References
1	Class Bivalvia	*Villorita cyprinoides*	*Argopecten ventricosus*	Cambrian-Terreneuvian	520–541	^100–102^
2	Superfamily Solenoidea	*Sinonovacula constricta*	*Solen strictus*	Lower Middle Triassic (Anisian, Carnian)	228–247	^103, 104^
3	Superfamily Tellinoidea	*Donax vittatus*	*Nuttallia olivacea*	Carboniferous—Upper Missisipian (Serpukhovian)	323–330	^105^
4	Superfamily Cardioidea	*Acanthcardia tuberculata*	*Tridacna squamosa*	Upper Ordovician (Porkuni)	444–446	^106^
5	Family Corbiculidae	*Villorita cyprinoides*	*Geloina coaxans*	Early Jurrasic-Hettangian-Aalenian	170–201	^68^

Early Jurrasic fossil record of *Corbicula* sp.(201–170 mya) was used to establish the primary appearance of Cyrenid clam^68^. According to the dated tree, diversification of Cyrenid clams from genus *Geloina* occurred roughly around early Jurassic, 179 mya (HPD, Highest Posterior Density: 169–198 mya) whereas, the evolutionary split between *Corbicula* and *Villorita* lineage appeared during early Cretaceous, 124 mya (HPD: 56–173 mya).

The splitting of vesicomyid clams is dated near to the early Cenozoic era i.e., 47 mya (HPD: 14–102 mya) corroborating previous investigation^69^. Also, the evolutionary radiation of Imparidentian bivalves is found to be in the Mesozoic Era in tune with preceding researches^57, 58^. The evolutionary radiations of major superfamilies from our study were found to be Solenoidea − 239 mya-, Tellinoidea − 328 mya-, Mactroidea − 233 mya- and Veneroidea − 268 mya.

## Conclusion

Even though enormous mitogenomes of clams have been characterised, there is dearth in molecular data for economically and ecologically significant clam, *Villorita cyprinoides*. The present study determined the first complete mitochondrial genome of multivalent endemic black clam, *Villorita cyprinoides*, which can be considered as keystone species that can perform top-down and bottom-up control^70^. Further, characterisation of mitogenome of *V. cyprinoides* resembles typical metazoan mitogenome architecture including the ATP8 gene, which is absent in several heterodont bivalves. Besides, conserved gene order (GO) analysis states the similarity of *V. cyprinoides* to *C. fluminea,* which is acceptable with detected phylogeny, revealing that both are closely associated and only recently diverged from an immediate common ancestor. The comparative GO analysis also revealed the conserved gene order of protein-coding and rRNA genes of *V. cyprinoides* and Vesicomid clams like *Archivesica* sp. and *C. magnifica.*

The present phylogenetic analysis using both mitochondrial and nuclear genes resolved the monophyletic status of family Cyrenidae confirming a sister relationship with the Arcticoid family. This study also estimated the diversification of Cyrenid clams during the early Jurassic-Cretaceous period. Resolving phylogenetic uncertainty and estimation of divergence time in family Cyrenidae is important, as it is a potential taxon to trace bivalve radiation to the inland water system because it retains both brackish water and freshwater genera^18^. Though we verified monophyly of Cyrenidae and enriched the transparency of phylogenetic status, integrated investigation of other Cyrenid clams with dense species sampling is required to conserve our conclusion and to provide evolutionary divergence among and between taxon.

Mitogenome sequences can be utilised for comparative phylogenomic studies within family Cyrenidae and development of genetic markers for identifying cryptic species if any. Apart from this, the genetic markers developed from this genome sequence can be used to study the population structure of *V. cyprinoides* since structure analysis is mandatory before the effective implementation of clam relaying programmes^71^. Even though no report on family Cyrenidae for the presence of an alternative mitochondrial inheritance pattern, Doubly Uniparental Inheritance (DUI)^72^, needs to be determined in the genus *Villorita*. At present, a reliable fishery-dependant data is available for stock assessment of black clam^18^ and the mitogenome characterised here has scope to provide genetic markers to have fishery-independent estimates of stock biomass. The findings shall provide a better base to evaluate if stock biomasses are at sustainable levels.

## Methods

### Specimen collection

Samples of *V. cyprinoides* were collected from Muhamma region in Vembanad Lake (approximate GPS coordinates: 9°36′18.6″N 76°22′01.2″E), Kerala, India at June 2017, transported and maintained alive in Central Marine Fisheries Research Institute (CMFRI, Kochi, India) hatchery. Specimens used in this study were identified by taxonomical literature^73, 74^ and COI barcoding^75^. Sex was determined by microscopic examination of the gonads. The report of heteroplasmy in male clams from several bivalve families^76^ prompted us to select a female specimen with the average size for tissue extraction. Tissue from adductor muscles was extracted and stored in absolute alcohol and RNA later (− 20 °C) in duplicate. After a day of tissue extraction, the samples stored in RNA later were transported in dry ice to Xcelris Genomics (Gujarat, India).

### Genomic DNA extraction, PCR amplification and sequencing

Total genomic DNA was extracted from ethanol fixed adductor muscle tissue using the Qiagen DNeasy Blood and Tissue Kit (QIAGEN, Valencia, CA, USA) and stored in − 80 °C. NANODROP ONE (Thermo Scientific) assessed the quantity and purity of the extracted DNA. Isolated genomic DNA served as a template for the amplification of nuclear genes (Histone H3, 18S rRNA). Both the genes were amplified in 25 µl reaction volume constituting 12.5 µl TaKaRa EmeraldAmp GT PCR Master Mix, 0.5 µl of 10 µM of each forward primer (18S rRNA: 5´ AACCTGGTTGATCCTGCCAGT 3´, H3: 5′ ATGGCTCGTACCAAGCAGACVGC 3′) and reverse primer (18S rRNA: 5′ GATCCTTCTGCAGGTTCACCTAC 3´, H3: 5′ ATATCCTTRGGCATRATRGTGAC 3′)^77, 78^ and 1 µl of 100 ng/µl DNA. PCR reaction was performed on BIORAD T100 TM thermal cycler (Biorad, USA) following the standard thermal regime with an annealing temperature of 55 °C for 18S rRNA and 52 °C for Histone H3 gene. The amplified product was purified and sequenced bidirectionally. Complementary sequences were visualised, edited and assembled into consensus sequence using BioEdit v7.1.9. The edited and assembled nucleotide sequences were deposited in GenBank.

### Mt-DNA isolation, library preparation, and sequencing

Mt-DNA was isolated and validated with mt-DNA specific genes for qualitative analysis. The concentration of the mt-DNA was measured using Qubit Fluorometer (Life Technologies, USA).

The paired-end sequencing library was prepared using QlAseq FX DNA Library Kit. 100 ng DNA was enzymatically sheared into smaller fragments. DNA fragments with an average length of 700 bp were end-repaired, 3′ adenylated and ligated to Illumina adapters. Adapter-ligated libraries were PCR amplified, quantification and size evaluation of the library was performed in Bioanalyzer 2100 using High Sensitivity DNA chip (Agilent Technologies) and sequenced on Illumina sequencing platform (Illumina HiSeq 2500, USA).

### Mitogenome assembly

Raw sequences generated after sequencing (2 × 150 PE) were retrieved in the FASTQ format. The quality of the reads was evaluated using FastQC software^79^. Illumina adapters, sequences less than 60 bp and low-quality sequences (Phred scores < 30) were removed using Trimmomatic^80^ leaving 17 million (PE) high quality reads. High-quality reads were subjected to De novo assembly using CLC workbench 6 (Qiagen, Germany) with a read map back algorithm. Similarity search of high coverage scaffolds (> 15kbps) was carried out against NCBI’s Nucleotide (NT) database using the BLASTN^81^ algorithm.

### Sequence annotation and analysis

The scaffold identified as mitogenome of *Villorita cyprinoides* was annotated using MITOS2^82^ web server substituting translation table 5. Besides MITOS2, Protein-Coding Genes (PCGs) were identified using the online ORF Finder Tool (https://www.ncbi.nlm.nih.gov/orffinder/). Ribosomal subunit (*rrn*S and *rrn*L) genes were confirmed using NCBI BLAST (https://www.nchi.nlm.nih.gov/BLAST/ ) searches. Validation of tRNA gene, structure prediction, and visualisation was accomplished with tRNAScan-SE v2.0^83^ and ARWEN v1.2^84^ under invertebrate mitochondrial settings. The cloverleaf structures of predicted tRNA genes were drawn using PseudoViewer v.3.0 (https://pseudoviewer.inha.ac.kr/)^85^ by sequence and structure input format and off numbering draw option. Control Region (CR) was annotated using DNA folding form from the Mfold web server (https://unafold.rna.albany.edu/?q=mfold/DNA-Folding-Form)^86^ retaining all the options as default except folding temperature (20 °C) and exterior loop-type (flat). Boundaries of MITOS2 annotated genes were refined based on the above software results using MEGA 7^87^. DNAPlotter^88^ was used to generate a circular visual overview of annotated mitogenome. The curated mitogenome sequence (Accession No: MK481950) was submitted to the NCBI database through the BankIt sequence submission tool (https://www.ncbi.nlm.nih.gov/BankIt/). Conserved gene order analyses of mt-genomes in superfamily level were conducted in superorder Imparidentia.

Nucleotide composition statistics of PCGs excluding stop codon and mitochondrial ribosomal RNAs were calculated separately by Geneious v11.1.5^89^. Codon usage bias and Relative Synonymous Codon Usage (RSCU) of protein-coding genes were inferred using the codon usage table computed through DnaSP v5.10.01^90^. AT and GC skew were analysed by the formula AT skew = (A + T)/(A-T) and GC Skew = (G + C)/(G−C)^91^.

### Phylogenetic reconstruction and divergence time estimation

The phylogenetic position of *V. cyprinoides* concerning superorder Imparidentia was appraised. For better-resolved molecular phylogeny, 47 bivalve mitogenomes belonging to 15 families from superorder Imparidentia and their available nuclear (18S rRNA and Histone H3) genes from GenBank database were used (Supplementary Table S5). The nucleotide sequence of all the 12 protein-coding genes excluding stop codons was used whereas the ATP8 gene was omitted from analysis owing to its lack in most of the bivalves. Besides coding genes, sequences of the small and large ribosomal subunit of mitogenome coupled with nuclear small ribosomal subunit and Histone H3 were incorporated. Seven species belonging to class Polyplacophora, Gastropoda and from order Pectenoida served as outgroups.

The amino acid sequence of 12 protein-coding genes and nucleotide sequences of *rrn*L, *rrn*S and nuclear 18S rRNA, Histone H3 genes were separately aligned using Clustal W^92^ implemented in MEGA7 with a default setting for PCGs. The aligned amino acid sequences of protein-coding genes were manually corrected and back-translated to nucleotide sequences. On the contrary, gap opening and extension costs for ribosomal gene alignments were set to 20/4 due to the high sequence variability in analysed taxa. After alignment, Gblocks v0.91b^93^ under default condition, changing the type of sequence, t = dna, identified and eliminated the ambiguous loci from mitochondrial and nuclear (18S) ribosomal RNA gene.

Appropriate subset partitions and evolutionary models for genes (Supplementary Table S4) were selected by PartitionFinder v.1.1.1^94^ under the greedy algorithm, unlinked branch length, and Bayesian information criterion (BIC) parameters. Protein coding genes and non-coding genes are considered as separate partitions. The best-fit model for partitions was chosen to perform Maximum likelihood (ML) and Bayesian Inference (BI). Phylogenetic analysis using the Maximum likelihood method was inferred using IQ-tree with 1,000 ultrafast bootstrapping replicates^95^. Bayesian analysis was implemented using MrBayes v.3.2^96^. The programme was run for 2 × 10^7^ generations comprising two simultaneous analysis with four chains, sampling every 100 generations. The average standard deviation of split frequency beneath 0.01 was considered as the stationarity phase, which is evaluated in Tracer v.1.6 (https://tree.bio.ed.ac.uk/software/tracer/), and the 50% majority rule summarised trees after discarding the first 25% as burn-in. Remaining trees were used to construct a consensus tree, edited and visualised in FigTree v.1.4.3 (https://tree.bio.ed.ac.uk/software/figtree/).

Estimation of divergence time between species and clade were determined using Bayesian relaxed clock models and MCMC algorithms^97, 98^ implemented in the mcmctree programme of PAML software v4.9^99^. While the rooted tree from the previous analysis was used to calibrate evolutionary rates by incorporating multiple fossil ages spanning from the earliest bivalve fossil to fossil from family Cyrenidae (Table 4) and parameters model = 4, alpha = 0.5, clock = 2 and rgene gamma = 2.2. Besides, a loose upper bound (maximal age constraint of 1,000 mya) was specified due to non-calibration at the root. The analysis was conducted with auto finetune for 20,000 iterations and the first 2000 iterations were discarded as burnin. The consensus tree was edited and visualised in FigTree v.1.4.3 (https://tree.bio.ed.ac.uk/software/figtree/ ).

### Ethics statement

No permission is required to collect and study the black clam, *Villorita cyprinoides*. They are not under an endangered or protected list and thus no control over the collection of samples.

### Consent for publication

The authors hereby declare that consent of the concerned individuals have been obtained to the publication of images Fig. 1b–h.

## Supplementary information

Supplementary information.
