# Supplementary material for: Mitogenomic architecture of the multivalent endemic black clam (Villorita cyprinoides) and its phylogenetic implications
Source: Sci Rep. 2020 Sep 22;10:15438. doi: 10.1038/s41598-020-72194-1 (PMC7508841; doi:10.1038/s41598-020-72194-1)
Supplement: Supplementary file 1 — Supplementary information. [file 41598_2020_72194_MOESM1_ESM.docx]

**Supplementary data**

**Mitogenomic architecture of the multivalent endemic Black Clam (*Villorita cyprinoides*) and its phylogenetic implications**

Summaya Rahuman^1, 3^, Jeena N. S^1^*, Asokan P. K^2^, Vidya R^2^ and Vijayagopal P^1^.

^1^Marine Biotechnology Division, Central Marine Fisheries Research Institute, Kochi, 682 018, Kerala, India.

^2^Molluscan Fisheries Division, Central Marine Fisheries Research Institute, Kochi, 682 018, Kerala, India.

^3^Mangalore University, Mangalagangotri, Mangalore, 574 199, Karnataka, India.

* Corresponding author’s E-mail: [jeenans@rediffmail.com](mailto:jeenans@rediffmail.com)

**Supplementary Table S1. Base composition, GC skews and, AT skews of complete mitogenomes used in this study.**

| **Species Name** | **A (%)** | **T (%)** | **G (%)** | **C (%)** | **GC (%)** | **GC Skew**  **G-C/G+C** | **AT Skew**  **A-T/A+T** |
| --- | --- | --- | --- | --- | --- | --- | --- |
| *Villorita cyprinoides* | 25.1 | 42.9 | 22.8 | 9.2 | 32 | 0.42 | -0.26 |
| *Arctica islandica* | 29.0 | 40.3 | 19.9 | 10.8 | 30.7 | 0.29 | -0.16 |
| *Acanthocardia tuberculata* | 24.6 | 35.4 | 23.5 | 16.6 | 40 | 0.17 | -0.18 |
| *Ceratoderma edule* | 23.9 | 34.3 | 23.9 | 17.8 | 41.8 | 0.14 | -0.17 |
| *Fulvia mutica* | 27.9 | 36.6 | 22.7 | 12.8 | 35.5 | 0.27 | -0.13 |
| *Hippopus hippopus* | 26.4 | 33.7 | 24.6 | 15.3 | 39.9 | 0.23 | -0.12 |
| *Tridacna derasa* | 28.4 | 36.9 | 21.9 | 12.7 | 34.7 | 0.26 | -0.13 |
| *Tridacna squamosa* | 27.4 | 35.0 | 22.3 | 15.3 | 37.6 | 0.18 | -0.12 |
| *Coelomactra antiquata* | 25.7 | 38.5 | 23.3 | 12.5 | 35.8 | 0.3 | -0.2 |
| *Lutraria maxima* | 22.7 | 41.2 | 25.1 | 10.9 | 36.1 | 0.39 | -0.28 |
| *Lutraria rhynchaena* | 22.3 | 40.0 | 26.3 | 11.4 | 36.7 | 0.39 | -0.28 |
| *Mactra chinensis* | 24.7 | 39.0 | 22.8 | 13.5 | 36.3 | 0.25 | -0.22 |
| *Donax vittatus* | 28.3 | 35.2 | 21.2 | 15.0 | 36.5 | 0.17 | -0.1 |
| *Donax variegatus* | 24.8 | 35.6 | 23.8 | 15.7 | 39.6 | 0.2 | -0.17 |
| *Donax trunculus* | 25.3 | 33.6 | 24.7 | 16.2 | 41.1 | 0.2 | -0.13 |
| *Donax semiestriatus* | 26.9 | 35.0 | 22.3 | 15.6 | 38.1 | 0.17 | -0.13 |
| *Moerella iridescens* | 25.5 | 40.3 | 23.2 | 11.1 | 34.2 | 0.35 | -0.22 |
| *Nuttallia olivacea* | 27.8 | 37.5 | 22.9 | 11.9 | 34.7 | 0.31 | -0.14 |
| *Semele scabra* | 22.7 | 36.5 | 29.1 | 11.7 | 40.8 | 0.42 | -0.23 |
| *Semelidae* sp. | 27.2 | 41.1 | 20.0 | 11.7 | 31.7 | 0.26 | -0.2 |
| *Sinonovacula constricta* | 25.9 | 41.1 | 22.5 | 10.5 | 33 | 0.36 | -0.22 |
| *Solecurtus divaricatus* | 21.3 | 38.8 | 27.5 | 12.3 | 39.9 | 0.38 | -0.29 |
| *Solen grandis* | 22.6 | 42.2 | 24.5 | 10.7 | 35.2 | 0.39 | -0.3 |
| *Solen strictus* | 21.7 | 40.9 | 25.6 | 11.7 | 37.4 | 0.37 | -0.3 |
| *Calyptogena magnifica* | 27.5 | 40.9 | 22.0 | 9.6 | 31.6 | 0.39 | -0.19 |
| *Pseudocardium sachalinense* | 24.3 | 35.1 | 24.9 | 15.7 | 40.6 | 0.22 | -0.18 |
| *Soletellina diphos* | 23.5 | 39.8 | 25.1 | 11.6 | 36.7 | 0.36 | -0.25 |
| *Cyclina sinensis* | 26.2 | 46.8 | 19.1 | 7.8 | 27 | 0.41 | -0.28 |
| *Dosinia japonica* | 28.2 | 41.8 | 20.9 | 9.1 | 30 | 0.39 | -0.19 |
| *Dosinia troscheli* | 28.1 | 41.6 | 21.3 | 9.0 | 30.3 | 0.4 | -0.19 |
| *Dosinia altior* | 27.8 | 41.8 | 21.8 | 8.6 | 30.4 | 0.43 | -0.2 |
| *Meretrix petechialis* | 25.3 | 43.0 | 22.0 | 9.6 | 33.5 | 0.39 | -0.25 |
| *Meretrix lamarckii* | 23.7 | 42.0 | 24.5 | 9.8 | 34.3 | 0.42 | -0.27 |
| *Meretrix lusoria* | 25.0 | 42.9 | 22.8 | 9.3 | 32.1 | 0.42 | -0.26 |
| *Meretrix lyrata* | 25.9 | 44.6 | 21.4 | 8.1 | 29.5 | 0.44 | -0.26 |
| *Meretrix meretrix* | 25.5 | 42.9 | 22.0 | 9.6 | 31.6 | 0.39 | -0.25 |
| *Paphia undulata* | 27.6 | 37.3 | 23.8 | 11.3 | 35.1 | 0.35 | -0.14 |
| *Paphia textile* | 27.5 | 36.8 | 24.5 | 11.2 | 35.7 | 0.37 | -0.14 |
| *Paphia amabilis* | 28.1 | 35.2 | 23.7 | 13.0 | 36.7 | 0.29 | -0.11 |
| *Paphia euglypta* | 28.5 | 38.4 | 22.3 | 10.8 | 33.1 | 0.34 | -0.14 |
| *Ruditapes phillipinarum* | 29.9 | 39.5 | 20.6 | 9.8 | 30.6 | 0.35 | -0.13 |
| *Ruditapes decussatus* | 27.9 | 35.1 | 23.7 | 13.3 | 37 | 0.28 | -0.11 |
| *Saxidomus purpuratus* | 25.8 | 40.4 | 22.8 | 11.0 | 33.8 | 0.34 | -0.22 |


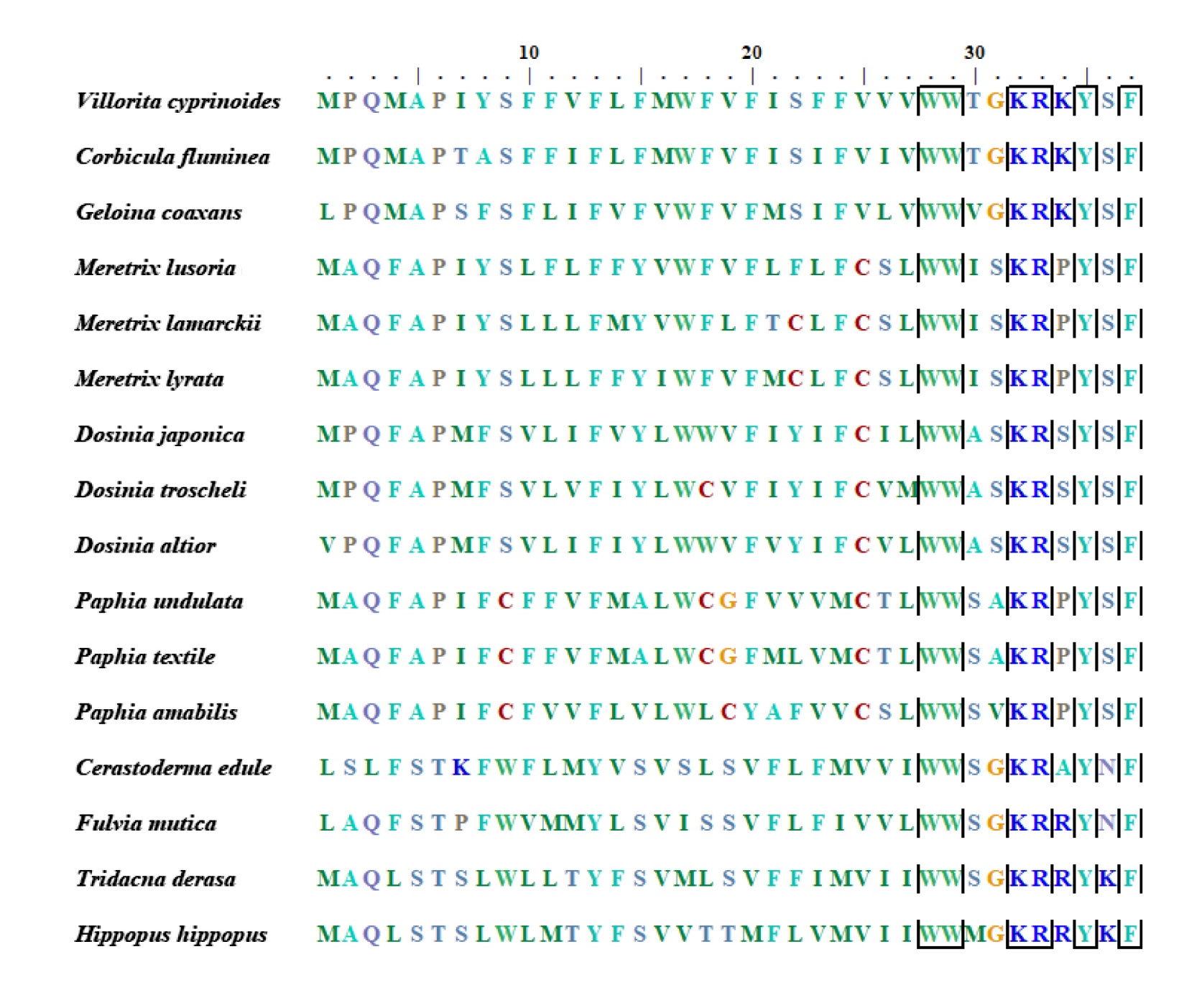
**Supplementary figure S2: Figure showing the alignment of conserved amino acid sequences of ATP8 gene in species belonging to family Cyrenidae, Veneridae, and Cariidae.** The conserved amino acid sequences were outlined.

**Supplementary figure S3: Putative secondary structure of the control region of *V. cyprinoides* inferred by the Mfoldweb server (http://unafold.rna.albany.edu/?q=mfold/DNA-Folding-Form). The Gibbs energy (dG) of the structure is shown at the bottom.**


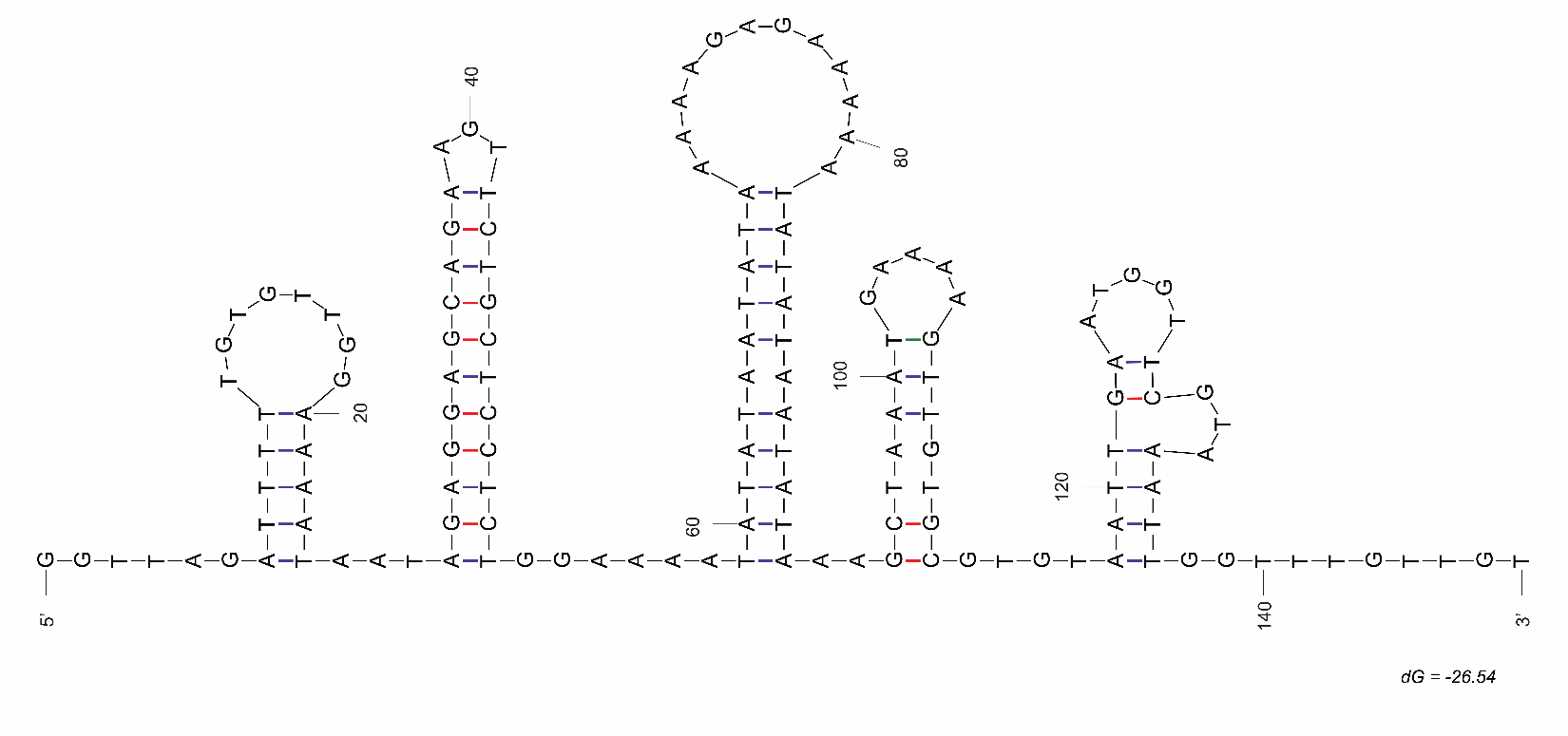


**Supplementary Table S4: The Best Partitioning Scheme and Evolutionary Model for Bayesian analysis generated by Partition Finder.**

| **Subset** | **Subset codon partition** | **Subset site** | **Best Model** |
| --- | --- | --- | --- |
|  | cox1pos1 | 1-1488\3 | GTR+I+G |
|  | cox1pos2 | 2-1488\3 | TVM+I+G |
|  | cox1 pos3, cox2pos3, cox3pos3, cytb pos3, nad1pos3 | 3-1488\3, 1494-2118\3, 2124-2916\3, 2922-4014\3, 4020-4872\3 | TIM+I+G |
|  | cox2pos1, cox3pos1 | 1492-2118\3, 2122-2916\3 | GTR+I+G |
|  | cox2pos2, cox3pos2 | 1490-2115\3, 2117-2910\3 | TVM+I+G |
|  | cytb pos1, nad1pos1, nad3pos1 | 2920-4014\3, 4018-4872\3, 5761-6048\3 | GTR+I+G |
|  | cytb pos2, nad1pos2, nad3pos2 | 2921-4014\3, 4019-4872\3, 5762-6048\3 | TVM+I+G |
|  | nad2pos1, nad4lpos1, nad6pos1, ATP6pos1 | 4876-5757\3, 7168-7434\3, 8713-9012\3, 9016-9675\3 | GTR+I+G |
|  | nad2pos2, nad4pos2, nad5pos2 | 4877-5757\3, 6053-7164\3, 7439-8709\3 | GTR+I+G |
|  | nad2pos3, nad4lpos3, nad6pos3 | 4878-5757\3, 7170-7434\3, 8715-9012\3 | HKY+G |
|  | nad3pos3, nad4pos3, nad5pos3 | 5763-6048\3, 6054-7164\3, 7440-8709\3 | HKY+G |
|  | nad4pos1, nad5pos1 | 6052-7164\3, 7438-8709\3 | GTR+I+G |
|  | nad4lpos2, nad6pos2, ATP6pos2 | 7169-7434\3, 8714-9012\3, 9017-9675\3 | TVM+I+G |
|  | ATP6pos3 | 9018-9675\3 | HKY+G |
|  | *rrn*S, *rrn*L | 9676-9843, 9844-10246 | TVM+I+G |
|  | 18S rRNA | 10247-11415 | TrNef+I+G |
|  | Histone H3 | 11416-11767 | GTR+G+I |

**Supplementary Table S5. List of species used for phylogenetic analysis.**

| **Order** | **Superfamily** | **Family** | **Species name** | **GenBank Accession no.** | | |
| --- | --- | --- | --- | --- | --- | --- |
|  |  |  |  | **Mitogenome** | **18S rRNA** | **Histone H3** |
| Venerida | Cyrenoidea | Cyrenidae | *Villorita cyprinoides* | *MK481950 | *MT459442 | *MT559334 |
|  |  |  | *Corbicula fluminea* | KX254564 | AM774558 | AY070161 |
|  |  |  | *Geloina coaxans* | KP999913 | - | - |
|  | Glossoidea | Vesicomyidae | *Calyptogena magnifica* | NC028724 | KC429381 | - |
|  |  |  | *Archivesica* sp*.* | MF959624 | KX010207 | - |
|  | Arcticoidea | Arcticoidae | *Arctica islandica* | NC022709 | AM774563 | DQ184901 |
|  | Veneroidea | Veneridae | *Cyclina sinensis* | NC029478 | EF426289 | HM124624 |
|  |  |  | *Meretrix petechialis* | NC012767 | - | HM124637 |
|  |  |  | *Meretrix lamarckii* | NC016174 | - | HM124633 |
|  |  |  | *Meretrix lusoria* | NC014809 | JN996714 | FJ429107 |
|  |  |  | *Meretrix lyrata* | NC022924 | JN996715 | FJ429109 |
|  |  |  | *Meretrix meretrix* | NC013188 | EF426291 | HM124634 |
|  |  |  | *Saxidomus purpuratus* | NC026728 | EF426294 | - |
|  |  |  | *Dosinia japonica* | NC038063 | JN996713 | HM124626 |
|  |  |  | *Dosinia troscheli* | NC037917 | - | HM124630 |
|  |  |  | *Dosinia altior* | NC037916 | - |  |
|  |  |  | *Paphia undulata* | NC016891 | JN996722 | HM124648 |
|  |  |  | *Paphia textile* | NC016890 | JN996721 |  |
|  |  |  | *Paphia amabilis* | NC016889 | JN996719 | HM124655 |
|  |  |  | *Paphia euglypta* | NC014579 | - | DQ184877 |
|  |  |  | *Ruditapes phillipinarum* | NC031332 | EF426293 | EF670667 |
|  |  |  | *Ruditapes decussatus* | NC035757 | - | DQ458531 |
|  | Mactroidea | Mactridae | *Lutraria maxima* | NC036766 | - | - |
|  |  |  | *Lutraria rhynchaena* | NC023384 | - | - |
|  |  |  | *Mactra chinensis* | NC025510 | EF583913 | - |
|  |  |  | *Coelomactra antiquata* | NC021375 | - | - |
|  |  |  | *Pseudocardium sachalinense* | MG431821 | - | - |
| Cardiida | Cardioidea | Cardiidae | *Acanthocardia tuberculata* | NC008452 | AM774522 | KR422700 |
|  |  |  | *Ceratoderma edule* | NC035728 | AY570555 | KC429217 |
|  |  |  | *Fulvia mutica* | NC022194 | D88911 | KR422761 |
|  |  | Tridacnidae | *Hippopus hippopus* | NC039944 | D84660 | - |
|  |  |  | *Tridacna derasa* | NC039945 | D84658 | - |
|  |  |  | *Tridacna squamosa* | NC026558 | D84190 | - |
|  | Tellinoidea | Psammobiidae | *Nuttallia olivacea* | NC018373 | - | MG517233 |
|  |  |  | *Soletellina diphos* | NC018372 | KX185515 | KU605620 |
|  |  | Semelidae | *Semele scabra* | NC018374 | - | - |
|  |  |  | *Semelidae* sp*.* | KX815956 | - | - |
|  |  | Solecurtidae | *Solecurtus divaricatus* | NC018376 | EF613233 | - |
|  |  | Tellinidae | *Moerella iridescens* | NC018371 | EF613237 | - |
|  |  | Donacidae | *Donax vittatus* | NC035987 | MF668494 | MF668473 |
|  |  |  | *Donax variegatus* | NC035986 | MF668497 | MF668473 |
|  |  |  | *Donax trunculus* | NC035985 | KC429395 | KC429226 |
|  |  |  | *Donax semiestriatus* | NC035984 | MF668493 | MF668464 |
| Adapedonta | Solenoidea | Pharidae | *Sinonovacula constricta* | NC011075 | - | - |
|  |  | Solenidae | *Solen grandis* | NC016665 | - | - |
|  |  |  | *Solen strictus* | NC017616 | AB714787 | AB714868 |
| Myida | Dreissenoidea | Dreissenidae | *Dreissena polymorpha* | KY091877 | JX099478 | KC429234 |

****This study***
